# An algorithmic perspective on deciphering cell–cell interactions with spatial omics data

**DOI:** 10.1093/bib/bbaf236

**Published:** 2025-05-25

**Authors:** Mike van Santvoort, Federica Eduati

**Affiliations:** Department of Mathematics and Computer Science, Eindhoven University of Technology, Groene Loper 3, 5612AE Eindhoven, The Netherlands; Institute for Complex Molecular Systems, Eindhoven University of Technology, Groene Loper 3, 5612AE Eindhoven, The Netherlands; Institute for Complex Molecular Systems, Eindhoven University of Technology, Groene Loper 3, 5612AE Eindhoven, The Netherlands; Department of Biomedical Engineering, Eindhoven University of Technology, Groene Loper 3, 5612AE Eindhoven, The Netherlands

**Keywords:** cell–cell interaction modeling, spatial omics, spatial data, statistical correlation, supervised learning, optimization

## Abstract

The advent of technologies to measure molecule information from a tissue that retains spatial information paved the way for the development of cell–cell interaction (CCI) methods. Even though these spatial technologies are still in their relative infancy, the developed methods promise more accurate analysis of CCIs due to the inclusion of spatial data. In this review, we outline these methods and provide a high-level view of the algorithms they employ. Moreover, we investigate how they deal with the spatial nature of the data they use and what types of downstream analyses they execute. We show that spatial CCI methods can broadly be classified into supervised learning, statistical correlation, and optimization methods that are used for either refinement of CCI networks, spatial clustering, differential expression analysis, or analysis of signal propagation through a tissue. In the end, we highlight some avenues for the development of complementary CCI methods that exploit advances in spatial data or alleviate certain downsides of the current methods.

## Introduction

Cells rarely function on their own. They coordinate together to execute tasks in your body. They achieve this through cell–cell interactions (CCIs), where cells send molecular messages to each other in order to alter their function. Understanding how cells communicate provides valuable insights into processes such as cell development [1], tissue homeostasis [2], and disease progression [3].

CCIs can be inferred from molecular data, particularly transcriptomics, coming from patient tissue. Traditionally, these data have been generated through bulk RNA sequencing, although nowadays more and more detailed information is leveraged through the maturation of single cell transcriptomics [4, 5].

This need to leverage ever more detailed information from a patient’s tissue led to the advent of new technologies measuring molecules at a spatial resolution. These techniques have been developed for transcriptomics [6], proteomics [7], metabolomics [8], and even multi-omics [9] and enabled the investigation of the spatial architecture [10] of patient tissue and brought a diversification of methods to infer CCIs (as reviewed in [5, 11]).

As more methods are developed to analyze CCIs using spatial omics, it is important to outline the mathematical techniques they employ. This helps to clarify their advantages, limitations, and optimal application. Existing reviews on spatial omics and spatial CCI models either focus on the direct analysis of spatial omics data [10, 12, 13] or describe spatial CCI models without emphasizing the mathematical features critical for CCI inference [5, 14]. Therefore, a review that highlights the broader mathematical aspects of spatial CCI modeling can provide valuable insights for selecting and developing CCI models tailored to specific use cases.

In this review, we provide an overview of CCI models and the role of spatial omics in them. We identify the algorithmic principles that drive these methods, categorize them based on their mathematical foundation, and discuss their potential advantages and limitations. Moreover, we examine how each method incorporates spatial information from their input data and how they leverage other contextual tissue information. Finally, we highlight the unique features that distinguish each method within their respective categories.

## Description of spatial omics data

Spatial omics techniques extract information about molecule expression from tissue while preserving spatial information. Classically, spatial omics techniques are subdivided into sequencing and imaging techniques [6, 10, 12, 15]. However, in the context of CCIs, it is more useful to classify them as spot based and identifier based, since, from a data perspective, these have to be dealt with differently. Spot-based techniques divide the tissue into regions (spots) and extract data from these regions, while identifier techniques detect molecular identifiers (like fluorescent labels [16]) from the tissue without relying on fixed regions [6, 14, 17].

Targeting spatial regions can be achieved in multiple ways. For example, one can physically remove the regions from the tissue, as is done in laser capture microdissection [18], these individual regions can then be subjected to, e.g. mass spectrometry, to extract information about lipids, metabolites, or proteins [19, 20]. Alternatively, one can add barcodes to all messenger RNA (mRNA) that appears in a specific region of the tissue, as in spatial transcriptomics [21]. Finally, targeting specific identifiers can, e.g. be achieved by attaching fluorescent tags to specific configurations of mRNA that encode for interesting proteins, as is done in smFISH [22].

**Figure 1:**
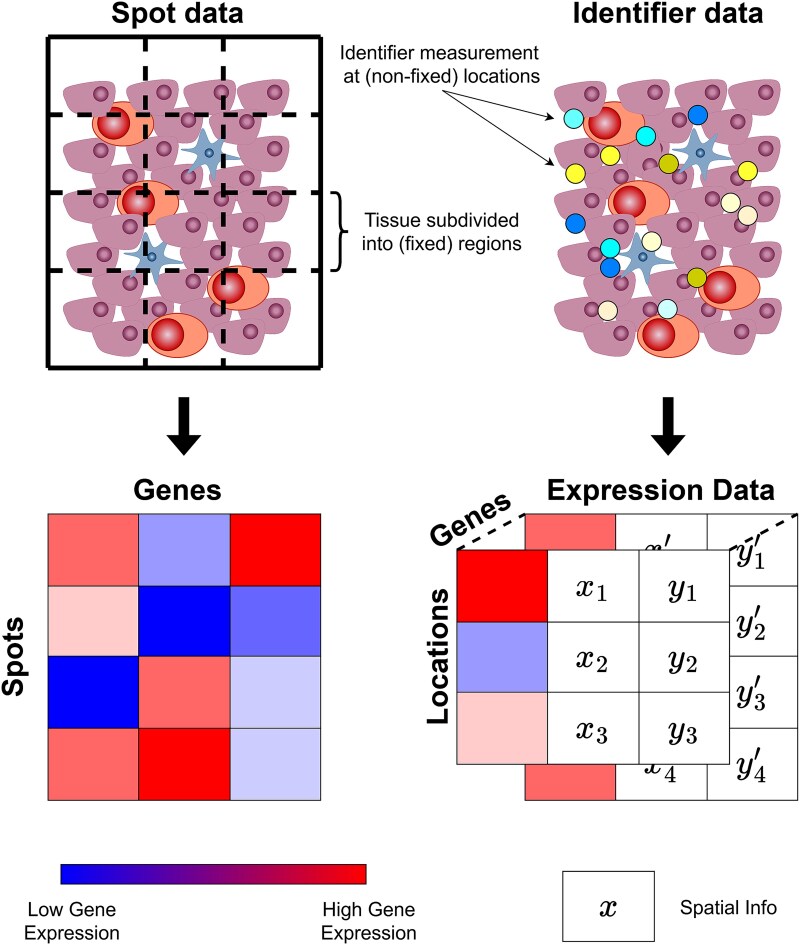
Architecture of spatial omics data. Note that the two types of data differ in how they deal with spatial information. Spot-based data have the same locations for all genes while identifier-based data can have different locations for different genes.

In spot-based approaches, data can be represented as a matrix where each row is a spot and each column a gene. The values in the matrix represent the expression of a fixed gene at a fixed spot [23]. For identifier-based techniques, data can be represented as a matrix per gene, with rows corresponding to locations (e.g. individual cells). The data in each column then represent the expression level and the coordinates of the protein (since these might be different for each gene) [24]. See Fig. 1 for a visualization.

The main difference between the two data types is that spot-based data provide discrete spatial information limited to the chosen spots, whereas identifier-based data offer continuous spatial resolution across the tissue. In general, due to the discrete nature of spot data, CCI methods operate most easily with spot data; however, identifier data can always be employed in conjunction with spot data by discretizing the continuous spatial region. If a method were to solely use identifier data, then it is impossible to apply it to spot data since it is unclear how to make spot data continuous in space.

As the name suggests, the primary advantage of spatial omics data is its ability to capture spatial information about a target tissue. This advantage is particularly important for CCI modeling since the addition of the spatial dimension provides important additional context [4] that models can leverage. In the remainder of this review, we will analyze this, starting with how spatial methods incorporate distance in their assumptions.

## Dealing with distance

CCI models that employ spatial data must account for the added dimension that it provides: the geometric aspect. They do this by making assumptions about how distance affects their interactions. For instance, ligands attached to the cell membrane (e.g. MHC-I [25]) are expected to interact only with nearby cells, whereas secreted ligands (e.g. IL-6 [26]) can mediate communications over greater distances.

There are two main approaches that CCI methods take to model how interactions are affected by distance. The first approach limits cell interaction to a fixed distance. In this case, a radius is chosen, and cells, proteins, or spots can interact only if their distance falls within this radius. This approach fits well with proteins on the cell membrane interacting through juxtacrine signaling [4]. CCI methods that adopt this approach include Copulacci [27], through its spatial adjacency graph, and MERINGUE [28], through its adjacency weight matrix. A second approach assumes the likelihood of an interaction decays when the distance between spots increases. This decay in interaction strength is often modeled as an exponential function, following protein diffusion principles [29], though other decay functions, such as linear decay, have also been used [30]. Note that long-distance signaling is never prioritized, presumably because tissue slides at the foundation of spatial data only encompass a small physical area.

Apart from general assumptions on the decay of interaction strength for increasing distance, CCI methods also differ in how they account for variations among protein classes. Most methods keep interaction decay over distance homogeneous for all proteins [30–32]. While this simplifies modeling, it can be biologically problematic since the optimal interaction distance might vary depending on the type of signaling [33]. Other methods indeed identify classes of proteins for which distance dependence differs, staying closer to the biological background [23].

However, from a practical perspective, keeping distance dependence homogeneous across all proteins simplifies modeling choices. It is difficult to exactly assess how distance dependence varies between molecule classes, and it is not always possible to assign a single type of signaling to every protein. For example, PD-L1 [34] can act both as surface bound and as secreted ligand [35], making it challenging to define a consistent interaction decay function. In such cases, a more homogeneous approach might perform better overall, even though it is less biologically accurate.

## Spatial data for cell–cell interaction validation and enhancement

Spatial omics data can be used to validate and enhance CCI models built from bulk or single-cell data. In validation, spatial data are used to confirm that predicted strong interactions between specific cell types or proteins correspond to their spatial proximity. This is done under the assumption that communication is stronger between colocated cells, in line with the idea that interaction only occurs within fixed boundaries or decays as distance increases. Methods like RaCInG employ this validation technique [36].

For enhancement, spatial data provide context on colocalization and tissue architecture, helping methods like BulkSignalR [37] and CellChat [38, 39] refine CCI predictions by incorporating spatial proximity to prioritize interactions between colocalized cells and adjusting predictions based on tissue structure.

Although these approaches are valuable, a downside is the gap between different data types. Spatial transcriptomics and single-cell or bulk RNA-seq cannot typically be performed on the same tissue section, as spatial methods require fixation and permeabilization, which degrade RNA, while single-cell and bulk RNA-seq require dissociation, destroying spatial context. As a result, these methods are often applied to adjacent sections or different regions, introducing potential discrepancies due to spatial heterogeneity [40].

## Cell–cell interaction inference using spatial omics data

**Figure 2:**
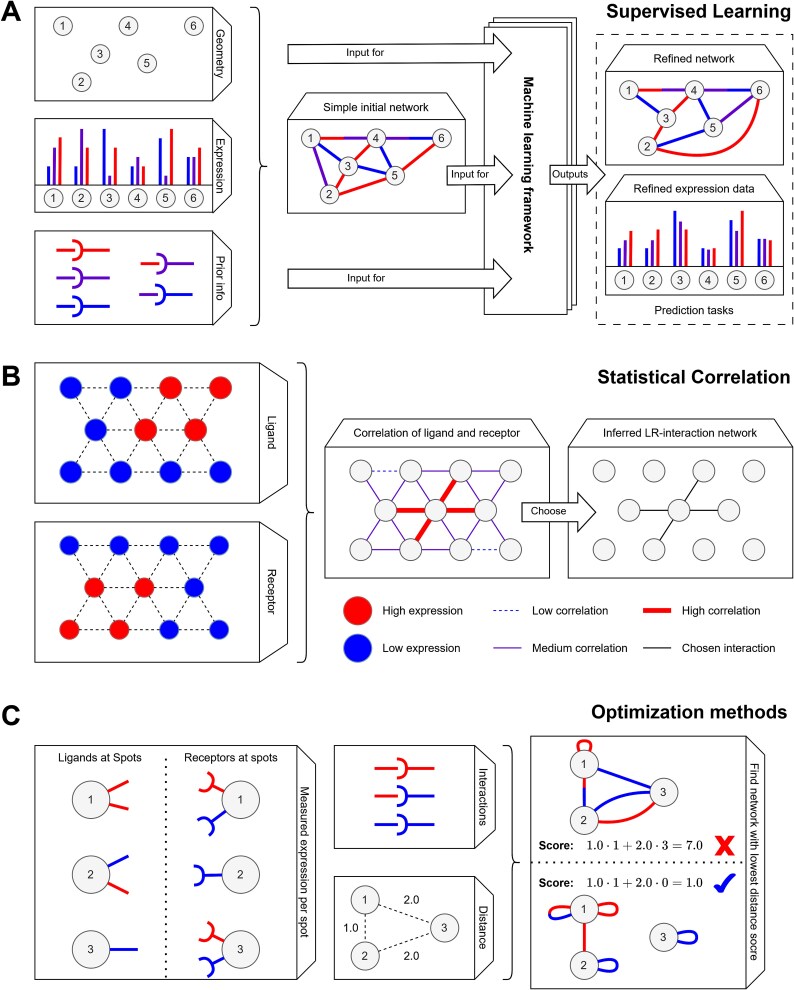
Visual overview of CCI inference methods using spatial omics. (A) Supervised learning methods, taking spatial coordinates of data, molecule expression, and prior knowledge to build an initial CCI network used for prediction/data refinement tasks through machine learning methods. (B). Statistical correlation methods, which compute a spatial correlation between ligand and receptor expression to build an interaction network for each LR pair. (C) Optimization methods that find the optimal interaction assignment, such that the most interactions take place over the least amount of distance.

Apart from merely serving as a validation or enhancement technique, spatial omics data can also serve as the foundation for CCI inference models. Such models aim to outperform models based on, e.g. bulk data. Methodologically, these methods can be broadly categorized into supervised learning methods, statistical methods, and optimization methods. See Fig. 2 for a visual overview.

In the section on dealing with distance, we already discussed what type of interaction decay assumption methods based on spatial omics might employ. However, each method may also vary in its specific algorithms, type of spatial omics data, and downstream analysis. Table 1 provides an overview of these distinctions. In this section, we focus on the methodologies themselves, leaving the downstream analysis to the next section.

**Table 1:** Overview of CCI methods that use spatial omics data analyzed for this review. For each method, it is outlined how the spatial omics data are structured (spot/identifier based), how the method deals with distance (i.e. the type of interaction penalty over distance assumed and whether or not protein classes are identified that deal differently with distance), what the general class of the method is, what type of interaction network is built, what types of CCI analyses can be done with the method, and finally, what one unique feature of the method is.

**Method**	**spOmic type**	**Distance assumption**	**Method type**	**Interaction type**	**Type of CCI analysis**	**Unique feature**
MIGGRI [24]	Identifiers	Uniform quadratic penalty for all proteins	Learning (GNN)	Gene interactions	Refinement	Based on images of expressed proteins.
HoloNet [23]	Spots	Varying exponential penalty per protein class	Learning (GNN)	Interaction contribution to expression	Refinement	Builds an interaction graph for each LR pair and uses this to inform the graph neural network.
NCEM [59]	Spots	Uniform maximum distance threshold for all proteins	Learning (GNN)	LR interaction	Refinement, differential expression	Layers of the GNN are updated based on full receptor expression at the target cell and full ligand expression at the neighboring cells.
SPACE [42]	Spots	Uniform maximum distance threshold for all proteins	Learning (GAE)	Spatially informed gene expression	Refinement, Spatial clustering	Uses graph attention networks rather than graph convolutional neural networks.
CLARIFY [31]	Spots	Uniform maximum distance threshold for all proteins	Learning (GAE)	Gene interactions	Spatial clustering	Builds an initial network on both the cell level and the gene level.
SpaGraphCCI [60]	Spots	Uniform maximum distance threshold for all proteins	Learning (GAE)	Cell–cell interactions	Differential expression	Is able to combine both gene expression per spot as well as additional pathological information.
MISTy [46]	Spots	Varying Exponential penalty, linear penalty, or maximum distance threshold per protein class.	Learning (Random Forest)	Interaction contribution to expression	Refinement, differential expression	Explainable machine learning model that can take information based on multiple tissue views.
SpatialDM [32]	Spots	Uniform exponential penalty + maximum distance threshold for all proteins	Correlation (coefficient)	LR interaction	Spatial clustering, differential expression	Changed permutation-based statistical test to find interacting LR pairs into a *z*-score approximation to improve efficiency.
MERINGUE [28]	Spots	Uniform maximum distance threshold for all proteins	Correlation (coefficient)	Cell–cell interactions	Spatial clustering	Direct adjacency of cells/spots is done through Voronoi tessellation instead of taking a ball with a fixed radius.
stLearn [70]	Identifiers + spots	Uniform maximum distance threshold for all proteins	Correlation (coefficient)	Cell–cell interactions	Refinement, spatial clustering	Is able to work on both spot-based spatial omics data and identifier based through a binning procedure.
Giotto [58]	Spots	Uniform maximum distance threshold for all proteins	Correlation (coefficient)	LR interaction & spatially informed gene expression	Spatial clustering	Can incorporate both spatial and single-cell data and compare the CCI results (on the cell-type level) based on both.
CellNeighborEX [71]	Spots	Uniform maximum distance threshold for all proteins	Correlation (coefficient)	Gene interactions	Differential expression	Focusses on differential expression between spots with similar spatial structure (homotypic) and different spatial structure (heterotypic)
SpaCcLink [72]	Spots	Different uniform maximum distance for each protein	Correlation (coefficient)	Gene interactions	Spatial clustering, differential expression	Combines a statistical correlation method with a preprocessing step, solving an optimal transport problem to determine LR interaction range.
Copulacci [27]	Spots	Uniform maximum distance threshold for all proteins	Correlation (Copula)	LR interactions	Differential expression	Uses distributions of gene expression that fit the sparsity of spatial omics data.
SVCA [50]	Spots	Uniform exponential penalty for all proteins	Correlation (Gaussian Process)	Interaction contribution to expression	Refinement	Explicitly models variations in gene expression data due to other sources than cellular interaction.
COMMOT [30]	Spots	Uniform linear penalty + maximum distance for all proteins	Optimization (linear)	LR interaction	Spatial clustering, Differential expression, Signal propagation	Only a flexible deterministic method. Can be easily adapted through the assumed constraints.
SpaOTsc [52]	Spots	Uniform linear penalty for all proteins	Optimization (nonlinear)	Cell–cell and gene interaction	Refinement, spatial clustering, signal propagation	First calculates an optimal mapping between single-cell derived data and spatial data before optimizing over the spatial domain.

### Supervised learning methods

Supervised learning methods for CCI inference (see Fig. 2A) generally take as input: (i) the spatial structure of the data, (ii) the molecule expression level (e.g. genes or proteins) at these spatial locations, and, in some cases, (iii) the prior knowledge about potential molecular interactions. These inputs are preprocessed and combined to train an algorithm that learns how these pieces of information best integrate in a network. Instead of learning a network directly from raw data, these machine learning methods are generally aimed either at making measured data more compatible with an existing network or at identifying instances where interactions might be missed.

The first subclass of these methods, including “Multi-Instance Graph neural network model for GeneRegulatory network Inference” (MIGGRI) [24] and HoloNet [23], uses graph neural networks (GNNs) to infer CCIs. These methods operate in two steps. Firstly, they transform their inputs into a graph structure that can be used in the second step to learn a target output. In the case of HoloNet, this target is identifying how much each cell type effectively communicates via ligand–receptor (LR) pairs. In the case of MIGGRI, the target is identifying previously unknown gene interactions.

HoloNet achieves its goals by first creating an initial weighted spatial interaction network for each fixed LR pair. The weight between two spots in this network is equal to the product of the measured expression values, multiplied by an exponential distance penalty. For each LR pair, this weighted network forms the basis of the GNN, and, per spot, the probability of it having a given cell type is embedded in it. By passing this information around the network (i.e. multiplying the inputted node embedding by the adjacency matrix), it can be measured what information each node receives from other cell types through the network. Finally, by computing a weighted sum of the results of each LR network, it can be concluded what proportion of cell-type gene expression is due to LR activity and what proportion is intrinsic to the cell type. The weights in this final sum are learned by comparing the combined LR and intrinsic cell-type expression to the known measured data (which should be the sum of the two outputs) and minimizing mean squared error.

MIGGRI takes a slightly different approach by working directly with *in situ* hybridization images rather than spot-based gene expression data. It first creates a gene interaction network based on known gene interactions. This network will form the basis of the GNN. However, first, information from each image needs to be embedded in each node. To ensure that this embedding conveys gene interaction information, images of genes and their target are paired and passed through the same convolution network. This network is trained such that there is low contrastive loss [41] between the embedding of a gene and its target. Finally, images are embedded at the associated gene using the trained convolution network and embeddings are passed through the initial gene interaction network. At a fixed node $i$, this is done by combining the embedding at gene $i$ with the average embedding at the neighboring genes. New gene interaction scores are predicted by taking the dot product of the output embeddings of the GNN. Note that, although the resulting graphs and predicted interactions are nonspatial, MIGGRI utilizes the spatial information to best embed the images and determine whether genes interact.

The second subclass of these methods, including SPACE [42] and CLARIFY [31] uses graph autoencoders (GAEs) for mapping graph-based input data to a lower-dimensional space. Both methods aim to refine the spatial data they get as input in order to remove noise and amplify important spatial patterns. Both methods begin by constructing a naive spatial graph by applying a *k*-nearest neighbor algorithm to the cells/splots. CLARIFY even constructs a second naive gene regulatory network (GRN) on each cell using CeSpGRN [43] These networks will be used by the GAE to encode the spatial omics data.

To encode, SPACE maps the adjacency matrix of its native graph and the gene expression data per cell/spot to a 10-dimensional latent representation. It does this by using a three-layer graph attention network. CLARIFY, meanwhile, uses two separate parallel encoders to encode the cell-level graph/data and the gene-level graph/data. Both of these encoders pass the cell- and gene-level data along the constructed networks (as is also done in GNN architecture). Finally, to ensure that the gene-level encoding can also be used at the cell level, its result is averaged (or concatenated) over all genes in the initial GRN to obtain the encoding at the cell level from gene-level data. Both SPACE and CLARIFY use the same general method to decode encoded data: graph information is decoded using an inner-product decoder, while gene information is decoded through a fully connected neural network.

To train both encoders/decoders, both methods try to minimize the reconstruction loss of the originally constructed graph. The rationale is that this is valuable spatial information (or prior knowledge in the case of CLARIFY’s GRN) that should be retained. Additionally, SPACE also tries to minimize the mean squared error between the reconstructed gene expression while CLARIFY aims to ensure that the reconstructed gene expression has a block diagonal structure to ensure that it aligns with the subdivision of the GRN over the cells.

The main benefit of GNNs and GAEs is their ability to learn complex, nonlinear relationships in the data, which are often difficult to capture by white-box models [44]. Moreover, these methods can work effectively with simple initial networks, progressively removing noise and faulty assumptions during training. However, their flexibility comes at the cost of interpretability [45]: these black-box models provide limited insight into how predictions are made, potentially hindering their generalizability to different datasets or biological settings.

One approach to address this limitation, as is done in MISTy [46], is to focus on explainable machine learning models. Through algorithms like random forests MISTy ensures that choices of the model can be checked after training [47]. MISTy positions itself similarly to MIGGRI and HoloNet. First, MISTy creates an initial spatial dependency graph at multiple view levels set by the user. For each of the view levels, this graph is constructed differently, e.g. at the intrinsic view level, no connections are made with neighboring cells/spots; it models the effect of a cell/spot itself on a marker, while the local view level only makes connections with neighboring cells/spots.

Given these initial graphs, the model is trained in two steps. First, for each view, a separate random forest is trained to map the gene expression data (and spatial dependency graph) to a single output that can predict a target gene expression. Then, in the second level, all views are linearly combined, and their linear coefficients are estimated using regularized linear regression. The performance of this process is tested and optimized using cross-validation on instances where the target gene expression is known.

Even though MISTy can show how it reaches decisions after training, it remains difficult to influence or guide the decision-making process during training. This means we cannot directly steer the model to align with specific biological mechanisms or expert knowledge in a transparent way as it learns. Therefore, while supervised learning models are powerful in prediction tasks, white-box mathematical approaches can be seen as complementary tools to achieve greater interpretability and biological alignment in CCI inference.

Computationally, the fact that supervised learning methods can pick up complex patterns makes them costly. Both GNN and GAE exhibit, in general, a quadratic time complexity in the number of nodes used in the underlying networks [48], especially since the methods discussed in this review use a complete weighted graph in their computations (e.g. HoloNet), decode based on a complete graph (e.g. CLARIFY), or use convolutional neural networks in part of their architecture (e.g. MIGGRI).

### Statistical correlation methods

Statistical correlation methods (see Fig. 2B) take the counts of a given set of ligands and receptors over all spots and seek to calculate the correlation between the two. Heuristically, if the expression of ligand $i$ at spatial location $t$ has a large correlation with the expression of receptor $j$ at location $s$, then it is likely that there is cellular interaction between location $t$ and $s$ via LR pair $\left(i,j\right)$. The challenge that these methods try to overcome is computing this spatial correlation coefficient for all quadruples $\left(i,j,t,s\right)$.

**Figure 3:**
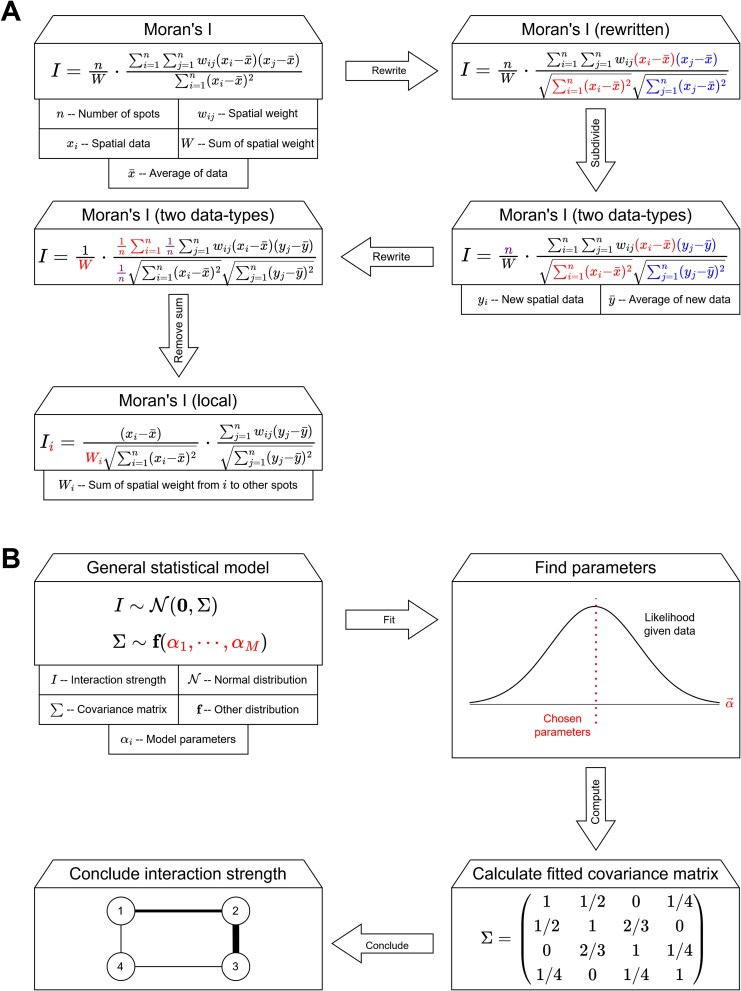
Statistical idea behind each subclass of statistical correlation method. (A) A schematic overview of how Moran’s *I* is transformed to obtain global spatial LR correlations (by assigning part of the formula to ligand contribution and part to receptor contribution) and local correlations (by removing one of the original sums in some way). (B) The general pipeline that Gaussian models employ.

To address this challenge, methods like SpatialDM [27] and MERINGUE [28] directly compute a spatial bivariate correlation coefficient from spRNAseq data. They do this by adapting Moran’s *I*, a spatial autocorrelation metric motivated by geography [49]. The main obstacle these adaptations need to overcome is enabling the metrics to use two types of data (e.g. the spatial expression of ligand $i$ and receptor $j$) rather than only one data type. This obstacle is usually overcome by assigning elements of the Moran’s *I* expression equally to both data types (see Fig. 3A).

By significance testing on the correlation coefficient, statistical correlation methods are able to identify which proteins are significantly (co-)expressed. In parallel, using a local version of Moran’s *I*, these methods are able to identify where these significantly (co-)expressed proteins interact. This local metric is usually constructed from Moran’s *I* by only summing over all spots $s$ from the perspective of a fixed spot $t$ (see Fig. 3A). Significance testing in this setting can be done by, e.g. permuting the assumed distance dependence between spots and taking the correlations in this setting as null distribution (MERINGUE) or approximating the distribution of spatial correlations by a normal distribution (SpatialDM).

Alternatively, it is also possible to model the joint expression of ligand $i$ at location $t$ and receptor $j$ at location $s$ as a random vector. Then, the correlation/covariance in this random vector can serve as the “strength” of an interaction. Copulacci [27] implements this approach through a Gaussian copula with Poisson marginals, while “Spatial Variance Component Analysis” (SVCA) [50] does this by fitting a Gaussian process model. The choice of a Gaussian copula/model is especially convenient since the correlation between protein distributions or spatial locations in the random vector can be retrieved through the Gaussian’s covariance matrix. In these models, parameters of the (Gaussian) covariance matrix and (Poisson) marginals (in case of Copulacci) are computed through maximum likelihood estimation, and the estimate of the parameter(s) in the covariance matrix determine protein interaction strength between two locations (see Fig. 3B for the general architecture).

The main benefit of statistical correlation methods is giving tight control over assumptions about CCIs. For example, if Poisson marginals do not model ligand and receptor expressions well, then the methodology can be adapted to use different marginal distributions. The main downside of these methods is that they compute LR interactions for one fixed ligand and receptor at a time, often disregarding the influence that the expression of one ligand might have on the expression of another ligand [51]. However, due to the white-box nature of these models, they could address this limitation if needed at the expense of additional computational complexity. For example, Copulacci could be adapted by construction of a multivariate Gaussian copula as opposed to a bivariate one. In this case, the covariance matrix would contain different correlation parameters for different LR pairs simultaneously (instead of one per copula) so that the expression of one LR pair can also influence another LR pair.

Computationally, correlation coefficient–based methods like SpatialDM are efficient in their deployment since a single deterministic expression has to be calculated for each LR or each spot. Hence, time complexity can be made linear if a maximum threshold on the radius of interacting spots is set. However, additional computational burden is introduced when testing for significance between spots: this is usually done through permutation tests, which are expensive for large datasets, although this can be alleviated by a *z*-score approximation [32].

The inference methods (like SVCA) suffer from a different computational burden. Since they need to estimate parameters of their underlying model, their bottleneck is usually the estimation procedure, the complexity of which scales with the number of parameters to be estimated. For these methods, this entails at least one parameter per pair of interacting spots. This is why these methods often heavily restrict where interactions can occur (like Copulacci) [27] or only estimate per spot the fraction of measured data that is explained by CCIs (like SVCA) [50].

### Optimization methods

A CCI network can be seen as a system where all spatial locations and protein types work together in an interacting system. In such a system, resources have to be shared and optimally allocated because the level at which resources are shared can be seen as the interaction level between cells or genes. Optimization methods (see Fig. 2C) align naturally with this perspective, as they aim to determine this allocation by minimizing the “effort” required to distribute measured protein/gene activity per spatial location to possible cellular interactions. In its simplest form, taken in the context of LR interactions from a set $I$ between spots from a set $S$, the objective function is as follows:


$$ \underset{X\in \varGamma }{\mathit{\min}}{\sum}_{\left(i,j\right)\in I}\ {\sum}_{\left(t,s\right)\in{S}^2}X\left(i,j,t,s\right)\cdotp d\left(i,j,t,s\right). $$

Here, $X\left(i,j,t,s\right)$ represents the interaction strength between the LR pair $\left(i,j\right)$ from spot $t$ to spot $s$. The goal of the optimization method is to compute these values such that their contribution over long distances is minimized. Here, this is represented by the distance penalty $d\left(i,j,t,s\right)$. Note that this penalty may vary between LR pairs, e.g. to differentiate between secreted and surface ligands. Often, a simple transformation of the Euclidean distance between spots is used, like squaring it [30].

Extra constraints can be added to the objective function through the set of admissible assignments $ \varGamma $. These constraints can be hard, e.g. ensuring that only LR interactions take place that have been previously observed in literature by setting $ X\left(i,j,\cdotp, \cdotp \right)=0 $ for all $ \left(i,j\right)\notin I $. However, they can also be softer, e.g. ensuring that a fixed proportion of measured activity of ligand $ i $ at spot $ t $, denoted by $ L\left(i,t\right) $, is assigned to interaction strength by requiring for fixed $ \alpha \in \left(0,1\right) $ that


$$ {\sum}_j{\sum}_sX\left(i,j,t,s\right)\ge \alpha \cdotp L\left(i,t\right). $$

No matter the specific constraints added to $\varGamma$ or additional penalties included in the objective function, they are always designed to align the optimization problem with the measured data and biological practice. For example, COMMOT [30] includes the aforementioned hard constraint as well as an additional penalty term for unassigned data, while SpaOTsc [52] includes a soft constraint, ensuring that the final values of $X$ cannot differ too much from the measured spatial data, nor from the measured single-cell omics data.

The main advantage of optimization methods compared to the two previously analyzed categories is that it is a white-box model that considers the genes, proteins, and cells in a tissue as an integrated interacting system. This is in contrast to the statistical models that usually only look at fixed protein pairs at the same time or machine learning models that cannot be steered as directly as optimization methods.

However, the strengths of the optimization methods are complementary to those of the other two categories of methods. One limitation of optimization methods is that they do not take into account stochasticity: they treat the input data as absolute rather than as potentially noisy and imprecise. This can be problematic for spatial omics data, where measured protein activity is often an imperfect representation of actual expression levels [53]. For example, SpaOTsc addresses this limitation by optimization by relaxing constraints to allow for slight deviations from the measured data, effectively accounting for variability in spatial measurements or between different data types.

Computationally, the discussed optimization methods are solved efficiently due to their formulation as convex problems. Therefore, they can leverage efficient iterative algorithms to find an approximate solution [54, 55]. The main computational burden of these methods lies in the fact that they are currently constrained in their efficiency by their convex formulation. Although such methods have also been applied in nonconvex settings, their performance is only guaranteed for convex problems [56, 57].

## Analyzing spatial cell–cell interaction networks

In the previous section, we focused on the algorithms behind spatial CCI models. See Table 2 for a general overview of their advantages and disadvantages, as well as points of note about computational complexity. In this section, we will highlight the types of CCI networks they generate and the downstream analyses they enable. We particularly highlight the ways in which the spatial dimension enriches insights that can be derived. See Table 1.

**Table 2:** General advantages and disadvantages of spatial omics CCI methods classes. For each method class, it is indicated what the general advantages and disadvantages of the class are as discussed in the review. Moreover, for each method the type of downstream analysis is indicated that methods within each class employ most and can execute best.

**Method class**	**Positive elements of modeling choice**	**Negative aspects of modeling choice**	**Source of computational burden**	**Best suited downstream analysis task**
Supervised learning	Can capture complex relationships that are difficult to capture by models that rely on explicit assumptions. Only requires a simple initial spatial structure that can be refined. Explicitly accommodates for noise.	Is a black-box model that cannot easily be manually steered.	The number of edges used in the underlying network (usually quadratic in the number of nodes).	Refinement, differential expression
Statistical correlation	Is a white-box model that can be easily adapted. Explicitly accommodates for noise.	Often deals with elements of measured data separately, removing the possibility of them interacting.	The number of possibly interacting spot pairs. The number of parameters to be estimated.	Spatial pattern clustering, differential expression
Optimization	Is a white-box model that can be easily adapted. Considers every aspect of measured data as an interacting system.	Does not explicitly consider the stochasticity of measured data.	Restriction to convex optimization formulation.	Signal propagation, differential expression

### Types of interactions

Even though all CCI models aim to use gene or protein expression data with spatial and cellular information to gauge how these cells interact, the nature of their output can differ. In some methods, the spatial or cellular component is mainly used during preprocessing or modeling but is no longer explicitly present in the final output. These models focus instead on inferring gene–gene interactions or refining gene expression profiles. Although they do not output CCIs directly, the resulting gene-level information is shaped by the spatial context. For example, MIGGRI [24] predicts gene interactions based on the expression of genes and their target in a tissue, while SPACE [42] and Giotto [58] create interaction-aware gene expression data.

There are other methods that do not produce explicit CCIs but instead model how gene or protein expression in a cell is shaped by both intrinsic factors and extrinsic influences from neighboring cells. By decomposing the expression into these components, such methods can reveal which genes or proteins partake most in intercellular communication and identify spatial regions with high interaction activity. Examples include HoloNet [23], SVCA [50], and MISTy [46], which enable downstream analyses of interaction intensity or spatial communication patterns without directly inferring specific CCIs.

Finally, a large group of methods explicitly infer CCIs. These can be split into methods that infer undirected interactions between cells and those that use prior knowledge of LR pairs to derive directed communication networks. For instance, NCEM [59] and SpatialDM [32] explicitly distinguish between ligands and receptors, while methods like MERINGUE [28] and SpaGraphCCI [60] do not. Often interactions are inferred between individual cells or spatial locations, although aggregate analyses on the level of cell types can also be executed by averaging over all spots/cells of a given type.

In general, methods that construct LR, cell–cell, or gene contribution to cellular interactions are *de novo*. These might use some initial knowledge on how, e.g. ligands and receptors can interact, but they infer the interactions between different spatial spots without the need of an initial spatial interaction structure. On the other hand, methods that infer gene interaction networks or refine gene expression data within the spatial context are based on an initial interaction structure that is used either to predict new interactions or refine the data in the context of this structure.

### Network and data refinement

One major application of spatial CCI models is to refine simple CCI networks using spatial data. This refinement can highlight specific spatial elements that explain observed phenomena. Machine learning methods, which often start with simple initial networks, are usually designed for and focus on this type of analysis to better understand the tumor microenvironment in specific tissue contexts.

For instance, HoloNet [23] refines the initial network it creates to explain what parts of its output (i.e. the refined gene expression data) are due to CCIs. In one case study, HoloNet focuses on MMP11 expression to explore its role in breast cancer. By analysing the refined gene expression data and especially the contribution of each gene to cell–cell communication, the method can predict where MMP11 expression takes place spatially and which LR pairs mediate MMP11 expression and determine which cell types actually communicate using this gene.

In this application, the inferred cell–cell communication network is not spatial in nature. However, this is not a limitation as spatial data provide a context that enhances the global understanding of cell and protein interactions. For example, MIGGRI [24] extends existing (global) gene interaction networks by identifying new potential interactions, which can be validated experimentally.

Other methods, like MISTy [46], combine multiple views (e.g. global and local) to refine the data by explaining how much of the expression of each measured marker (gene or protein) can be explained and predicted by each of the views. For instance, MISTy was able to capture a set of markers best explained by a global perspective rather than a local perspective. Potentially, such methods can offer insights into when spatial omics data have the most impact. Methods like MIGGRI [24] go even further, enabling the reconstruction of entire pieces of missing information, such as tissue images, to predict where genes might be expressed in the absence of spatial data.

Supervised learning methods are best suited for network and data refinement, due to their ability to learn complex patterns from data. For this task, their black-box nature is an advantage since models are not constrained by assumptions baked into the models. This gives them more freedom to remove types of noise even if they are not known beforehand.

### Spatial pattern clustering

Another valuable use of spatial CCI methods is to identify spatial patterns in their inferred interaction networks, such as groups of proteins that all operate in the same region. Clustering LR interactions into spatial groups provides insights into the role of protein in a specific context. The statistical inference methods have this type of analysis typically baked into their pipeline.

For example, spatialDM [32] clusters LR interactions into groups of spatially similar interactions, as demonstrated in a melanoma study, where it identified interactions specific to the lymphoid region. Such clustering provides contextual insights into interaction networks, helping identify treatment strategies tailored to a specific tumor context.

Apart from classifying inferred interactions into distinct spatial groups (based on their location), it is also possible to classify regions of the spatial tissue into classes. For example, CLARIFY [31] uses its refined gene expression data to identify spatial regions that “behave” similarly. These clustered spatial regions correlate well with distinct cell types, offering a better understanding of tissue. This enables, e.g. tracking tumor progression mapping immune infiltration levels.

Although all methods can employ spatial pattern clustering, the statistical class is especially suited for it. This is because the contribution of each LR or gene pair is directly encoded into spatial correlations between two spots. By constructing a distribution for each pair of spatial locations over all these values and contrasting them between locations, it is then possible to identify parts of the tissue that behave in a similar fashion. In this way, the methods turn their main downside into an upside: only ‘because’ correlations are often computed for fixed gene pairs separately (without any relation being assumed between them), they can be seen as independent samples of an underlying distribution.

### Signal propagation

Spatial CCI models also allow tracking how a signal of a certain gene or protein propagates through the tissue spatially. In this way, researchers can identify which parts of the tissue might provide the source of a certain signal, offering potential therapeutic targets for unwanted signals.

For example, SpaOTsc [52] uses the inferred CCI network to track how Wnt and bone morphogenetic protein (BMP) signaling occurs in zebrafish embryos. It pinpointed the region of signaling activity, identified the direction of signaling propagation, and constructed a distribution on the range of Wnt signaling. Such findings can be used, e.g. to update the distance assumption that the model makes or identify the importance of the direct neighborhood of a cell on signaling.

Optimization methods are particularly well suited for this task since their formulation of the objective function explicitly models CCIs as an optimal allocation of measured mass to the spatial structure of the data (i.e. an optimal transport problem). Therefore, by investigating what mass is assigned to parts of the tissue and seeing how this mass decays or strengthens in certain directions enables one to see how a signal moves through a tissue.

### Differential expression analysis

Finally, spatial CCI methods also enable differential expression analysis. Because spatial data include geometry as an inherent factor, spatial features can be used as metadata elements to compare inferred interactions. One could take interactions between different spatial regions as a type of metadata, or one could compare how given LR pairs interact at close distance versus long distance.

For example, COMMOT [30] identifies spatial patterns in its inferred CCI networks and uses them to analyze how signaling within cells differ spatially. Using the differential expression analysis, COMMOT managed to identify groups of genes that are indicative of a spatial cluster, enhancing functional understanding of cell clusters.

More traditional differential expression analysis, which does not consider the tissue’s geometry as a meta-feature, is also possible on the inferred CCI networks. For example, SpatialDM [32] compares LR expression across groups, such as between adults and fetal intestines, revealing spatial features changes across conditions. While traditional differential expression analysis is possible, it is more difficult in the context of spatial omics because large datasets are often unavailable and expensive to produce [15]. However, this problem will likely fade as spatial omics techniques commercialize and mature [61].

When these large datasets are available, all three classes of models are able to effectively do differential expression analysis. Of course, the specific features used will change depending on the output of a given model, statistical methods for example using the correlation values while supervised learning methods using the updated gene expression matrix. However, if the datasets are large enough, there is no reason to assume that either of the methods would perform better in this regard than the others.

## Conclusion

Spatial omics is a rapidly evolving branch of omics technologies, distinguished by its ability to capture the geometry of a tissue in its measurements. This capability gives CCI methods critical spatial heterogeneity information, enabling predictions that are more accurate, context driven, and biologically insightful. Spatial omics not only provides the opportunity to validate existing CCI methods, but it also opens the door for new CCI methods that leverage on the additional information that spatial omics data can provide.

Each CCI method differs in the exact spatial omics data it uses, the way it assumes interactions to decay at longer distances, its differentiation between protein types, and its global approach. As highlighted in this review, each method comes with its own strengths and limitations, making it essential to carefully evaluate which approach aligns best with specific data and research questions.

No matter the method employed, despite the promise of additional context through spatial data, it must also be highlighted that spatial data have unique drawbacks. First, spatial omics data can exhibit high variability in measured molecule expression due to tissue composition rather than true biological differences [62]. For example, certain molecules might appear highly expressed in a region simply due to local tissue heterogeneity, such as an abundance of certain cell types, whereas regions with sparse cell populations might falsely seem to lack significant expression. This variability complicates the inference of CCIs, as it introduces noise unrelated to true molecular signaling.

Second, spatial omics technologies are still maturing and often do not achieve the resolution or depth of single-cell omics [40]. There is a trade-off between spatial resolution and the number of molecules that can be simultaneously measured [61]. For example, in imaging-based techniques, the number of detectable proteins is limited by the amount of available distinct color channels to differentiate signals [63, 64]. Finer spatial resolution requires broader separation between colors, limiting the number of channels available [14]. Similarly, mass spectrometry methods used to measure metabolites and lipids are limited by the mass range they can measure [65, 66], being only semiquantitative if this issue is not present [8, 19].

While this limitation can be overcome by performing multiple rounds of imaging, merging data across rounds introduces new challenges such as ensuring alignment and consistency between images [62]. As spatial omics technologies evolve, advances like the development of more varied markers [61], techniques with higher resolution [67], and improved data integration methods will help mitigate these challenges, enabling broader and more detailed applications in CCI modeling. These techniques already exist to a certain extent, but are currently still very expensive to integrate into a modeling pipeline [8, 67, 68].

As the field of spatial omics continues to mature, advancements in data acquisition and processing are expected to further enhance the capabilities of CCI methods and alleviate the previously discussed drawbacks to some extent. Improved techniques for producing spatial data will not only help existing CCI methods reach their full potential but also pave the way for entirely new approaches. For example, a promising direction is the integration of time-dependent spatial omics data, which could enable tracking the temporal dynamics of disease progression [69]. Current CCI methods lack temporal analysis capabilities, presenting a significant opportunity for innovation in this area.

Even with the current state of spatial omics data, there are challenges that highlight the need for methodological advances. First, even though we have analyzed a broad range of models and highlighted some elements of computational burden, a full benchmark comparing the models is not available, possibly due to the fact that, often, models are built for varying data types. Such an extensive benchmark could further enrich the space of available comparisons between methods to aid in model choice and design.

Second, we have seen that supervised learning methods are not transparent in how they construct decisions, statistical correlation methods do not consider all protein types to (potentially) influence each other, and optimization-based methods do not consider possible noise in the spatial omics data. These gaps underscore the need for white-box models that consider all proteins and cells as components of an interacting system while also respecting the stochastic nature of the spatial omics data. Addressing these challenges will be crucial for the continued growth and utility of spatial CCI models in advancing our understanding of tissue biology and disease mechanisms.

**Key Points**

- Spatial-omics data provide an opportunity for cell–cell interaction (CCI) methods since the added spatial dimension can yield invaluable insight into tissue structure.
- Spatial CCI methods model interaction decay of molecules over distance, usually either by truncating possible interaction distances or by assuming smooth decay of interaction strength over distance.
- CCI methods using spatial omics can largely be classified into three groups: supervised learning, statistical correlation, and optimization.
- Each spatial CCI model excels at its own type of downstream analysis. Supervised learning methods often excel at data refinement, while statistical correlation methods excel at clustering spatial patterns.
- Even though a large body of spatial CCI methods have been developed, there is still room for further development alleviating the main issues with current methods or incorporating ever developing data types.

## Data Availability

None to declare.
